# Metabolic Constellations, Clusters, and Renal Function: Findings from the 2013–2018 National Health and Nutrition Examination Surveys

**DOI:** 10.3390/life11090904

**Published:** 2021-08-30

**Authors:** Kathleen E. Adair, Kelly R. Ylitalo, Jeffrey S. Forsse, LesLee K. Funderburk, Rodney G. Bowden

**Affiliations:** 1 Department of Health, Human Performance, and Recreation, Robbins College of Health and Human Sciences, Baylor University, Waco, TX 76798, USA; jeff_forsse@baylor.edu (J.S.F.); leslee_funderburk@baylor.edu (L.K.F.); 2 Department of Public Health, Robbins College of Health and Human Sciences, Baylor University, Waco, TX 76798, USA; kelly_ylitalo@baylor.edu

**Keywords:** metabolic syndrome, metabolic constellations, metabolic clusters, chronic kidney disease, renal function

## Abstract

Metabolic syndrome (MetS) is associated with decreased renal function and chronic kidney disease (CKD). To date, no research regarding the sixteen possible constellations resulting in the diagnosis of MetS has been elucidated. The purpose of this study is to report renal function in sixteen metabolic constellations grouped into four metabolic clusters. Individuals (n = 2767; representing 86,652,073 individuals) from the 2013–2018 National Health and Nutrition Examination Surveys who met the criteria for MetS were included. Sixteen possible constellations of three or more risk factors were analyzed for renal function. Four metabolic clusters representing MetS with hyperglycemia (Cluster I), MetS with hypertension (Cluster II), MetS with hyperglycemia and hypertension (Cluster III), or MetS with normoglycemia and normotension (Cluster IV) were assessed for renal function and CKD status. Cluster III had the highest odds of CKD (OR = 2.57, 95% CL = 1.79, 3.68). Clusters II and III had the lowest renal function and were not different from one another (87.82 and 87.28 mL/min/1.73 m^2^, *p* = 0.71). The constellation with the lowest renal function consisted of hypertension, high triglycerides, and a large waist circumference (82.86 mL/min/1.73 m^2^), whereas the constellation with the highest renal function consisted of hyperglycemia, low HDL, and a large waist circumference (107.46 mL/min/1.73 m^2^). The sixteen constellations of MetS do not have the same effects on renal function. More research is needed to understand the relationship between the various iterations of MetS and renal function.

## 1. Introduction

Metabolic syndrome (MetS) is a clustering of three or more interrelated metabolic risk factors including abdominal obesity, two dyslipidemia criteria including high triglycerides and low high-density lipoprotein (HDL) cholesterol, elevated blood pressure, and impaired fasting glucose [1,2]. Within the past three decades, the prevalence of individuals with MetS has increased from 25.3% to 34.2% in the United States (US) [3], trending with obesity, which has increased from 31.9% [4] to 42.6% [5] of the US population in the same time period. The diagnosis of MetS is predictive of cardiovascular disease (CVD) [6] and type 2 diabetes mellitus (T2D) [1,2], but it has also been associated with stroke [7], all-cause mortality [8], and chronic kidney disease (CKD) [9,10].

Metabolic syndrome and the individual risk factors associated with it pose risks to renal function [9,10]. The kidneys are vascular organs that are directly affected by weight gain [9] and the risk factors associated with metabolic diseases [10]. The process leading to CKD, and ultimately end-stage renal disease (ESRD), is characterized by the progressive and permanent scarring of the kidneys over time, known as glomerulosclerosis. Type 2 diabetes mellitus, the primary risk factor for CKD in the developed world [11,12], causes arterial stiffening [13,14,15], dilation of the afferent arterioles of the kidney’s glomeruli [16], and constriction of the efferent arterioles [13], resulting in a high pressure system in the glomeruli of the kidneys. Hypertension is the second leading cause of CKD in the developed world [11,12] and is mechanistically similar to T2D. Hypertension is brought about by arteriosclerosis, or hardening of the arteries, which causes reduced blood flow to the kidneys and renal ischemia. The renin-angiotensin-aldosterone system (RAAS) is engaged as a compensatory mechanism to increase blood flow to the kidneys but results in exacerbation of HTN and glomerulosclerosis [17].

The criteria established by the National Cholesterol Education Program’s (NCEP) Adult Treatment Panel III (ATP III) require the concurrent presence of at least three of the five metabolic risk factors to formally diagnose MetS. The combinations of three or more risk factors can result in 16 possible iterations of MetS, known as metabolic constellations. While each unique combination results in a diagnosis of MetS, it is possible that the various iterations have different etiologies and increase the risk of different outcomes, with some being more synergistic [6] than others in promoting disease outcomes. A study by Khosravi et al. [18] categorized the 16 metabolic constellations into four metabolic clusters, which were designed to emphasize hyperglycemia, hypertension (HTN), hyperglycemia and HTN, or normoglycemia and normotension (see Table 1). Khosravi et al. found that the cluster emphasizing HTN (cluster II) was highly associated with ischemic heart disease and CVD whereas the cluster that included both hyperglycemia and HTN (cluster III) was most closely associated with stroke [18].

To the best of our knowledge, the four metabolic cluster categories outlined in the study by Khosravi et al. [18] have never been analyzed for association with renal function. Given that hyperglycemia and HTN are the primary causes of CKD in the developed world, this unique analysis may assist in further elucidating the synergistic [6] effects of the metabolic risk factors and their associations with renal function. The purpose of the following study is to analyze and report renal function in each of the metabolic constellations and clusters in a representative sample of US adults. We hypothesize that the metabolic clusters will not be equally associated with renal function, and that those with HTN will have the lowest renal function.

## 2. Materials and Methods

This study was considered exempt from review by the sponsoring university’s institutional review board. Datasets were merged from the 2013–2014, 2015–2016, and 2017–2018 cycles of NHANES. Data were acquired from the Center for Disease Control and Prevention (CDC) website to be used in the present analyses. Survey sample weighting was conducted using a complex, four-stage, probability cluster from the National Center for Health Statistics Estimating and Weighting Procedures documents [19,20]. Sample weights are assigned to each subject, allowing researchers to extrapolate the results to be representative of all non-institutionalized US civilians. The NHANES study routinely oversamples underrepresented populations, including those 60 and over, African Americans, Asians, and Hispanics. Weighting takes into account the known probability of selection, non-responders, and the differences between the sample and the US population as a whole. The sample weighting is conducted in three steps. The first accounts for the oversampling of minority groups, the second adjusts for non-responders, and the third was a post-stratification matching the sample to a known civilian from the non-institutionalized US population, as determined by information from the US Census Bureau [21].

### 2.1. Study Sample

The criteria for the study included participants of the 2013–2014, 2015–2016, and 2017–2018 NHANES survey cycles. Individuals were required to be between the ages of 18 and 80 and have complete information to classify MetS (fasting blood glucose, fasting triglycerides, HDL, resting blood pressure, and waist circumference) and CKD (age, sex, and race). Only those with MetS were chosen for this study. The upper age limit was set because age is top coded at 80 years of age by NHANES for subject deidentification. We excluded individuals who were pregnant at the time of the study and those who were using dialysis in the 12 months prior to the study. A total of 2767 individuals met the study inclusion and exclusion criteria. Using the NHANES survey sample weighting techniques, this sample was representative of 86,652,073 non-institutionalized civilians living in the U.S.

### 2.2. Definition of Metabolic Risk Factors

The metabolic risk factors were defined using the National Cholesterol Education Program’s (NCEP) Adult Treatment Panel III (ATP III) [2]. Individuals were considered obese if they had a waist circumference (WC) > 101.6 cm for non-Asian males, >88.9 cm for non-Asian females, >94 cm for Asian males, or >80 cm for Asian females. Prescription medication information was classified using the International Classification of Diseases, Tenth Revision (ICD-10) codes [22]. Medications prescribed for hyperglycemia (R73, E11, E11.2, E11.2P, E11.4, and E11.P), hypercholesterolemia (E78.0, E78.0P, and E78.1), and HTN (I10 and I10.P) were taken into account in the present study. Hyperglycemia was classified as a fasting blood glucose ≥ 100 mg/dL or a prescription for glucose-lowering medication. Dyslipidemia was classified based on two criteria; the first of which was a fasting triglyceride level ≥150 mg/dL or prescription medication, and the second was a high-density lipoprotein-cholesterol (HDL) measurement <40 mg/dL for males or <50 mg/dL for females or prescription medication for dyslipidemia. Hypertension was classified as a resting systolic blood pressure >130 mmHg or a resting diastolic blood pressure >85 mmHg or a prescription medication for HTN.

### 2.3. Definition of Metabolic Constellations and Clusters

Metabolic constellations were defined as all possible combinations of 3 or more metabolic risk factors that could be used to diagnose MetS, resulting in 16 possible constellations. Metabolic clusters were modeled after the study by Khosravi et al. [18] where the constellations were categorized into 4 distinct groups, with an emphasis on hyperglycemia (Cluster I), HTN (Cluster II), hyperglycemia and HTN (Cluster III) or normoglycemia and normotension (Cluster IV). Cluster I included 4 subgroups of subjects with at least 3 metabolic risk factors, including hyperglycemia. Cluster II included 4 subgroups of subjects with at least 3 metabolic risk factors, including HTN. Cluster III included 7 subgroups of subjects with at least 3 metabolic risk factors, including hyperglycemia and HTN. Cluster IV included 1 subgroup of subjects with at least 3 metabolic risk factors, excluding hyperglycemia and HTN (see Table 1).

### 2.4. Outcome Measures

Estimated glomerular filtration rate (eGFR) was calculated using the Chronic Kidney Disease Epidemiology Collaboration (CKD-EPI) equation, [23] which has been demonstrated to be more accurate in predicting eGFR in individuals with higher values than the Modification of Diet in Renal Disease (MDRD) equation [23]. Chronic kidney disease was defined as an eGFR < 60 mL/min/1.73 m^2^ and/or an albumin to creatinine ratio (ACR) ≥ 30 mg/g [24].

### 2.5. Demographic and Biochemical Information

Trained interviewers conducted interview-style questionnaires using the computer-assisted personal interview (CAPI) system to determine demographic, socioeconomic, and lifestyle information for NHANES participants. The poverty index indicates the ratio of family income to poverty as defined by the Department of Health and Human Services (HHS). This index is commonly used as a criterion for determining eligibility in federal assistance programs [21]. A value of 1.0 indicates that an individual is at 100% of the poverty level, whereas a value of 2.0 indicates that an individual is at 200% of the poverty level. The exercise guidelines from the PA Guidelines Advisory Committee Report [25] were used to determine if subjects participated in ≥150 min of moderate-intensity recreational PA per week, ≥75 min of vigorous-intensity recreational PA per week, or an equivalent combination of the two. Individuals who met these criteria were considered physically active [25,26]. The smoking variable indicated if individuals had smoked at least 100 cigarettes in their lifetime or if they have used cigarettes in the past 5 days [27]. Individuals not meeting these thresholds were considered non-smokers. The prescription medication information used to classify metabolic risk factors was determined using the International Classification of Diseases, Tenth Revision (ICD-10) codes. The ICD-10 codes identified for this study were indicated for treatment of hyperglycemia (R73, E11, E11.2, E11.2P, E11.4, and E11.P), hypercholesterolemia (E78.0, E78.0P, and E78.1), and HTN (I10 and I10.P). NHANES physical examinations were conducted in a mobile examination center (MEC) and included anthropometric measures, blood pressure, blood panels and urinalysis.

### 2.6. Statistical Analysis

All statistical analyses were conducted in SAS version 9.4 (SAS Institute Inc., Cary, NC, USA). Complex survey sample weighting techniques established by NHANES were used for the analysis [19,20]. The sample weighting allows for extrapolation of the data to be representative of the greater U.S. population. In each of the analyses with weighted results, sample weights were incorportated by using the PROC SURVEY function in SAS. Three variables were assigned using the commands CLUSTER, STRATA, and WEIGHT: (1) a cluster variable known as the primary sampling unit (variable name SDMVPSU) (2) a stratification variable (variable name SDMVSTRA), and (3) a subsample weight (variable name WTSAF2YR). A DOMAIN statement was used in all analyses to include only the desired subsample. Unweighted continuous variables were presented as mean and standard deviation (SD) and unweighted categorical data was presented as frequency and percentage (%). Weighted continuous variables were presented as mean and standard error of the mean (SE) and weighted categorical variables were presented as percentage and standard error of percent (SE). Simple regression was used to determine the difference between weighted cluster values in the case of continuous variables. Chi square (χ^2^) tests were used to determine if there was a difference between the frequencies reported for each of the categorical demographic variables. The χ^2^ test does not indicate which group is different, but rather, indicates if there is a difference among any of the four clusters. A linear regression analysis was conducted where the outcome was eGFR and the regressor variables were the categorical cluster categories (Clusters I, II, III, and IV). The logistic regression analysis was used to determine odds of CKD in each of the four clusters. The assumptions of multiple regression were found tenable using histograms and Q-Q plots. The level of significance was set a priori at α = 0.05.

## 3. Results

A subsample of 2767 individuals representing 86,652,073 non-institutionalized US civilians with MetS was analyzed in the present study. The demographic data for the unweighted and weighted samples are in Table 2. Due to oversampling of individuals 60 and over, African Americans, Asians, and Hispanics, the unweighted sample is slightly older in age and more racially and ethnically diverse than the weighted sample. The four metabolic clusters represent individuals with MetS who have hyperglycemia without HTN (Cluster I), HTN without hyperglycemia (Cluster II), hyperglycemia and HTN (Cluster III) and normoglycemia and normotension (Cluster IV). The cluster with the greatest weighted frequency was Cluster III (62.08%) followed by Cluster I (24.71%). Cluster IV (3.66%) was least frequent in this population, followed by Cluster II (9.55%).

Cluster I had the highest frequency of Mexican Americans (16.13%) and a high average WC (110.44 cm), though all clusters demonstrated a WC that would qualify as a metabolic risk factor (110.70 cm). Cluster I also demonstrated a poor lipid profile with the second-highest TG value (182.31 mg/dL) and second-lowest HDL value (42.21 mg/dL) in the study sample. Still, this group demonstrated the second highest eGFR (97.67 mL/min/1.73 m^2^). Cluster II was predominantly female (57.4% female), had the highest frequency of NH Blacks (17.46%), and demonstrated the second lowest eGFR (87.82 mL/min/1.73 m^2^). Individuals in cluster III were more likely to be male (54.25% male) and were 20 years older, on average, than those in cluster IV. Individuals in Cluster III had the lowest socioeconomic status at 209% of the poverty level, which equates to an approximate income of $27,000/year for an individual or $55,385/year for a family of four. This cluster also demonstrated the highest WC (111.60 cm), ACR (64.78), and lowest eGFR (87.28 mL/min/1.73 m^2^) in the sample. Cluster IV was predominately female (56.18% female), demonstrated the poorest lipid profile with a fasting TG level of 228.30 mg/dL and a low HDL level at 37.16 mg/dL. However, this cluster demonstrated the best renal function represented by a high eGFR (106.44 mL/min/1.73 m^2^) and a low ACR (15.35).

The results of the regression analyses are presented in Table 3. In the linear regression analysis, renal function in each of the clusters was assessed. Clusters I and IV demonstrated the highest renal function whereas clusters II and III, which both included HTN, demonstrated the lowest renal function. In post-hoc testing, clusters 2 and 3 were not found to be statistically different from each other (*p* = 0.71). All other comparisons were significantly different at the *p* < 0.01 level of significance. The logistic regression analysis reports the odds of CKD for each of the clusters, with Cluster I as the reference. CKD was defined as an eGFR < 60 mL/min/1.73 m^2^ and/or an albumin to creatinine ratio (ACR) ≥ 30 mg/g [24]. Cluster III was the only cluster that was significantly different from the referent group, and individuals in this group had the highest odds of CKD (OR = 2.57, 95% CL = 1.79, 3.68, *p* < 0.001).

The frequency of CKD in each of the metabolic clusters is reported in Table 4. There were 19.22% that met the criteria for CKD, the majority of which were in cluster III (76.76%). Cluster III was the largest subgroup, approximately one quarter of which had CKD (23.76%). Cluster II was significantly smaller than Cluster III, representing only 8.31% of CKD cases, but it demonstrated a higher prevalence of individuals with CKD (16.73%) than the national average [11]. Cluster I was the second largest group, accounting for 13.91% of all CKD cases. However, this cluster demonstrated a lower proportion of CKD in the cluster (10.82%) than clusters II and III. The smallest cluster, cluster IV demonstrated the lowest frequency of CKD (5.30%).

**Figure 1:**
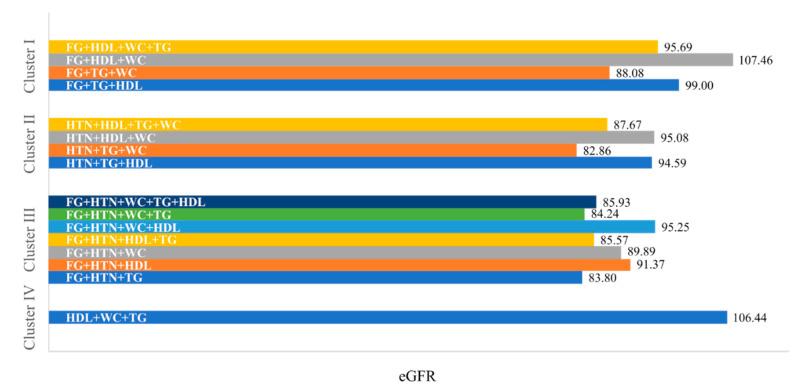
Renal function in the metabolic constellations, categorized by cluster. Data presented as eGFR (mL/min/1.73 m^2^) for each of the metabolic constellations. Cluster 1: MetS with hyperglycemia, Cluster 2: MetS with HTN, Cluster 3: MetS with hyperglycemia and HTN, Cluster 4: MetS with normoglycemia and normotension. Abbreviations: HTN, hypertension; FG, high fasting glucose; TG, high fasting triglycerides; HDL, low high-density lipoprotein; WC, high waist circumference; HTN, hypertension. The sum of weighted observations in this subsample was 86,652,073.

The renal function for each metabolic constellation, grouped by cluster, is represented in Figure 1. The constellations with the lowest renal function were HTN + TG + WC (82.86 mL/min/1.73 m^2^, n = 111), FG + HTN + TG (83.80 mL/min/1.73 m^2^, n = 180), and FG + HTN + WC + TG (84.24 mL/min/1.73 m^2^, n = 469). These constellations shared HTN and low TG as risk factors. The constellations with the highest renal function included FG + HDL + WC (107.46 mL/min/1.73 m^2^, n = 195), HDL + WC + TG (106.44 mL/min/1.73 m^2^, n = 91), and FG + TG + HDL (99.00 mL/min/1.73 m^2^, n = 64). These constellations shared HDL as a risk factor.

## 4. Discussion

In the present research study, we analyzed the renal function in individuals with metabolic syndrome to determine if there were differences in the various constellations and clusters of the disease. We found that the constellations with HTN and high triglycerides trended towards low eGFR whereas those with low HDL trended towards higher eGFR. The clusters associated with lowest renal function were Clusters II and III. Renal function was not found to be statistically different between these two clusters. The findings confirmed our hypotheses that there was a statistical difference in renal function between the four clusters and that HTN had a negative effect on renal function.

The study by Khosravi et al., which we used to model the metabolic constellations and clusters, was similar in sample size and age compared to our study, but the distribution of metabolic clusters was different. Our findings indicated that a majority of the individuals in the US who have MetS were in Cluster III (hyperglycemia and HTN), whereas the study by Khosravi et al. demonstrated a majority of their sample was classified into Cluster II (HTN with normoglycemia). The large variation in sample distribution across the four clusters could be due in part to the locations from which the samples were taken. Compared to the US population, the Iranian population in the Khosravi et al. study has a lower obesity rate (42.6% [5] and 22.7% [28], respectively) which may explain the lower proportion of individuals with hyperglycemia, since insulin resistance is frequently prompted by obesity [29].

We categorized metabolic constellations into four metabolic clusters which emphasized hyperglycemia, HTN, both hyperglycemia and HTN, or normoglycemia and normotension. In the study by Khosravi et al., Clusters II and III were found to be at highest risk of ischemic heart disease (IHD), CVD, and stroke. Similarly, our linear regression model demonstrated the lowest renal function in Clusters II and III, indicating a similar pattern of metabolic dysregulation. Our logistic regression model demonstrated the highest odds of CKD in Cluster III, which agrees with both the physiological [29] and epidemiological [11,12] research that indicates T2D and HTN as the main risk factors associated with CKD.

When looking specifically at the constellations associated with disease, Khosravi et al. demonstrated that the constellations most highly associated with IHD, CVD, and stroke were FG + HTN + TG, FG + HTN + HDL, and FG + HTN + HDL + TG, respectively. We found that renal function was lowest in the HTN + TG + WC constellation, classified in Cluster II. In our sample, the common denominators in the 3 constellations with lowest eGFR were HTN and high TG, whereas the common denominator in the 3 constellations with highest eGFR was low HDL. Similarly, high TG was found to be associated with renal dysfunction in the Atherosclerotic Risk in Communities (ARIC) study [30]. However, the ARIC study [30] and others [31,32] have found low HDL to be associated with renal decline, which is not consistent with our findings. It is possible that this phenomenon could be explained by the unique sample chosen, which only includes individuals with MetS. Low HDL may be the least predictive of renal dysfunction. Previous findings have indicated that individuals with a rare impairment of the scavenger receptor BI may have high levels of HDL, yet increased risk of coronary heart disease [33]. Additionally, individuals with extremely high levels of HDL have been shown to be at greater risk for all-cause mortality [34]. Further research should be done to better understand these, and other paradoxical findings related to high HDL levels.

The subsample of the US population that was analyzed in this study only included individuals with MetS, which is known to have a bidirectional relationship with CKD [9,10]. The proportion of CKD in this population was 19.22%, which is slightly higher than the 15% [11] reported for the US population. The higher rates of CKD were only seen in Clusters II and III, whereas clusters I and IV demonstrated lower than average proportions of CKD. Despite having MetS, individuals in Cluster IV had a higher eGFR and a lower proportion of CKD cases (5.30%) compared to the average population. Since this study is the first of its kind, this may be an indicator that certain configurations of MetS are less detrimental to renal health than others. Specifically, the constellation with low HDL, high WC, and high TG may not play as crucial of a role in the pathophysiology of CKD. However, this condition may be transient due to the younger age factor (37.40 years) of Cluster IV and its corresponding pattern of abdominal fat over time, which leads to the dysregulation of hormones, cytokines, and the development of insulin resistance [2,29].

One of the significant challenges with CKD epidemiology and treatment is that it goes largely undiagnosed [11]. There is an absence of signs and symptoms for the disease and many individuals are not tested or diagnosed as part of routine medical care due to the misperception that renal function declines due to older age. Nine out of every 10 adults with CKD are unaware that they have it, and one in two adults with very low kidney function do not know they have CKD [11]. One way to improve the early identification and prevention of CKD is to identify risk at earlier ages. A better understanding the etiology of the disease and the risk factors associated with renal decline are needed. Identifying the unique metabolic constellations that are most closely associated with CKD may aid in establishing screening procedures for medical reporting systems, which will enable physicians and medical staff to begin prophylactic treatment for declining renal function before it progresses to a critical stage.

The predominant characteristics of MetS are insulin resistance and abdominal obesity [2]; studies have demonstrated that MetS directly links to atherosclerotic CVD and T2D [1,2]. MetS has also been linked to CKD [9,10]; however, we established that there might be more harmful amalgamations of MetS to the kidneys than others. We saw a 24.6 mL/min/1.73 m^2^ range in eGFR between the highest and lowest renal function among the 16 constellations that were analyzed. We also established a wide variation in frequency of CKD, with 23.76% of Cluster III demonstrating the criteria for CKD and only 5.30% of Cluster IV demonstrating the criteria for CKD. Many iterations of MetS have been assessed in cross-sectional and longitudinal studies, resulting in a wide range of disease outcomes and risk associations [27,35,36,37]. The present study supports previous research findings of high variability of outcomes reported with MetS, indicating that not all constellations or clusters are equally detrimental to renal function. While this study was limited to a cross-sectional sample of non-institutionalized US citizens, it typifies new means for predicting and preventing CKD. Future research efforts should focus on the connection between metabolic constellations, clusters, and renal health.

### Strengths and Limitations

No studies have evaluated the relationship of metabolic constellations or clusters and CKD. To the best of our knowledge, this is the first study to analyze metabolic constellations and clusters with reference to renal function. The use of NHANES complex survey sample weighting techniques allowed us to extrapolate the results to the greater US population. Prior research in this area has been primarily in an Iranian population. The large datasets provided by NHANES were representative of a racially and ethnically diverse population. The study is limited by its cross-sectional nature, which precluded us from making causal inferences regarding the development of renal dysfunction. The sample sizes of the four clusters varied widely which lowered the statistical power of the study and made comparisons difficult between constellations and clusters. The primary measure of renal function was calculated using an equation for estimating glomerular filtration rate. This measure is highly influenced by serum creatinine values, which can be affected by hydration status, muscle mass, nutrition, exercise, medication, and muscle breakdown. While we did control for individuals who were pregnant and/or on dialysis, we did not control for drugs that may affect renal function due to the extensive drug list. Medications prescribed for hyperglycemia, hypercholesterolemia, and HTN were considered in the present study. Additionally, diagnosis of CKD should use two measurements of eGFR, separated by three months. Due to the cross-sectional nature of the study, we were unable to identify chronicity of disease.

## 5. Conclusions

The present study demonstrated that the diagnosis of MetS varied widely in prevalence and renal outcomes based on the clustering of risk factors. Many US citizens in this subsample had both hyperglycemia and HTN (Cluster III), and this cluster demonstrated the lowest renal function with the highest odds of CKD. The cluster with HTN and normoglycemia (Cluster II) also had low renal function, statistically equivalent to Cluster III. There was a 24.6 mL/min/1.73 m^2^ range in eGFR between the highest and lowest renal function among the 16 constellations that were analyzed. The three constellations with the lowest renal function shared HTN and high triglycerides as metabolic risk factors, whereas the three constellations with the highest renal function shared low HDL as a risk factor. These findings indicate that the 16 possible constellations which lead to the diagnosis of metabolic syndrome may play varying roles in the pathophysiology leading to renal decline. Clinicians can utilize the findings in this study to support patient health by identifying individuals at greatest risk for renal decline and CKD. Future research studies should aim to better understand the complex relationship between the metabolic constellations, clusters, and renal function. Additionally, longitudinal assessments are warranted to determine how metabolic constellations change over time and how their chronicity may affect renal function.

## Figures and Tables

**Table 1:** Criteria for metabolic clusters.

	Cluster I	Cluster II	Cluster III	Cluster IV
Constellations	FG, TG, HDL	HTN, TG, HDL	FG, HTN, TG	HDL, WC, TG
	FG, TG, WC	HTN, TG, WC	FG, HTN, HDL	
	FG, HDL, WC	HTN, HDL, WC	FG, HTN, WC	
	FG, HDL, WC, TG	HTN, HDL, WC, TG	FG, HTN, HDL, TG	
			FG, HTN, WC, HDL	
			FG, HTN, WC, TG	
			FG, HTN, WC, TG, HDL	

Cutoff values for all metabolic risk factors were outlined by the NCEP ATP III 2005 Revision [2]. Cluster groups were adapted from Khosravi et al. [11]. WC, large waist circumference; FG, high fasting glucose, or hyperglycemia; HTN, high blood pressure, or hypertension; TG, high triglycerides or dyslipidemia; HDL, low high-density lipoprotein or dyslipidemia, second separate criteria. Cluster 1: MetS with hyperglycemia, Cluster 2: MetS with HTN, Cluster 3: MetS with hyperglycemia and HTN, Cluster 4: MetS with normoglycemia and normotension.

**Table 2:** Demographic information for the subsample and metabolic clusters.

	Total		Cluster				*p*-Value
	Unweighted Total (n = 2767)	Weighted Total (n = 86,652,073)	Cluster I (24.71%)	Cluster II (9.55%)	Cluster III (62.08%)	Cluster IV (3.66%)	
Male Sex	1364 (49.3)	51.23 (1.30)	48.07 (3.16)	42.60 (4.30)	54.25 (1.78)	43.82 (6.03)	0.033
Age (years)	54.81 (14.62)	53.16 (0.52)	45.78 (0.91)	52.82 (1.00)	57.08 (0.50)	37.40 (1.45)	<0.001
Race/Ethnicity							
Mexican American	460 (16.62)	9.53 (1.08)	16.13 (1.79)	5.80 (1.59)	7.35 (1.10)	11.67 (3.11)	<0.001
Other Hispanic	320 (11.56)	5.54 (0.73)	6.44 (1.08)	3.23 (0.98)	5.35 (0.78)	8.64 (2.49)	
NH White	1020 (36.86)	65.77 (1.88)	65.29 (2.82)	67.14 (3.70)	66.02 (2.11)	61.10 (6.15)	
NH Black	603 (21.79)	11.10 (1.18)	4.59 (0.97)	17.46 (2.59)	12.78 (1.34)	9.98 (3.10)	
NH Asian	260 (9.40)	4.09 (0.42)	3.10 (0.57)	0	4.59 (0.51)	3.22 (1.57)	
Other/Multi-Racial	104 (3.76)	3.98 (0.49)	4.43 (0.91)	0	3.91 (0.75)	5.39 (2.95)	
Meets PA Rec **	720 (62.28)	60.34 (1.86)	58.28 (5.58)	65.46 (5.43)	60.37 (2.35)	62.42 (9.11)	0.849
Current Smoker	1388 (50.16)	52.08 (1.53)	51.85 (2.69)	48.09 (4.49)	53.06 (1.79)	47.38 (5.45)	0.577
Poverty Index	2.41 (1.59)	2.89 (0.06)	2.75 (0.12)	2.81 (0.16)	3.02 (0.08)	2.09 (0.16)	<0.001
BMI (kg/m^2^)	32.78 (7.03)	33.11 (0.27)	33.35 (0.45)	31.90 (0.51)	33.23 (0.26)	32.47 (0.72)	0.04
FG (mg/dL)	125.57 (46.83)	121.83 (0.95)	119.44 (1.76)	93.20 (0.39)	128.83 (1.42)	93.80 (0.60)	<0.001
SBP/DBP	131/72	129/73	117/69	133/76	135/74	114/70	<0.001
WC (cm)	109.25 (15.14)	110.70 (0.55)	110.44 (0.91)	106.75 (1.36)	111.60 (0.53)	107.41 (1.63)	<0.001
Fasting TG (mg/dL)	156.85 (153.88)	159.07 (3.62)	182.31 (7.85)	162.38 (8.10)	145.23 (3.26)	228.30 (13.01)	<0.001
HDL (mg/dL)	47.56 (14.56)	47.36 (0.42)	42.21 (0.67)	47.13 (1.07)	50.05 (0.54)	37.16 (0.65)	<0.001
SCr	0.89 (0.30)	0.88 (0.01)	0.83 (0.02)	0.90 (0.01)	0.90 (0.01)	0.81 (0.03)	<0.001
BUN	14.74 (6.02)	14.67 (0.18)	13.40 (0.25)	13.70 (0.36)	15.51 (0.21)	11.50 (0.38)	<0.001
Uric Acid	5.86 (1.49)	5.85 (0.04)	5.63 (0.07)	5.80 (0.08)	5.95 (0.05)	5.74 (0.18)	0.002
eGFR ml/min/1.73 m^2^	90.21 (21.97)	90.60 (0.72)	97.67 (1.37)	87.82 (1.33)	87.28 (0.76)	106.44 (2.27)	<0.001
ACR	69.28 (416.42)	48.96 (6.53)	23.97 (5.55)	23.18 (7.72)	64.78 (10.82)	15.35 (5.20)	0.003

Unweighted categorical data presented as n (%), continuous as mean (SD). Weighted categorical data presented as % (SE), continuous as mean (SEM). Cluster I: MetS with hyperglycemia, Cluster II: MetS with HTN, Cluster III: MetS with hyperglycemia and HTN, Cluster IV: MetS with normoglycemia and normotension. Abbreviations: HTN, hypertension; NH, non-Hispanic; PA, physical activity; BMI, body mass index; FG, fasting glucose; SBP/DPB, systolic blood pressure/diastolic blood pressure; WC, waist circumference; TG, triglycerides; HDL, high-density lipoprotein; SCr, serum creatinine; BUN, blood urea nitrogen; eGFR, estimated glomerular filtration rate; ACR, albumin to creatinine ratio; MetS, metabolic syndrome; SD, Standard deviation; SE, standard error; SEM, standard error of the mean. The *p*-value indicates the probability of observing a difference between the clusters for each variable. ** Missingness composed 41.78% of the physical activity variable. Values needed for creatinine, urea, and uric acid.

**Table 3:** Regression analyses of renal function (eGFR) in metabolic clusters.

	Linear Regression			Logistic Regression		
Coefficient	b	SE_b_	*p*-Value	OR	95% CL	*p*-Value
Cluster I (reference)	97.67	1.37	<0.001	-	-	-
Cluster II	−9.84	1.47	<0.001	1.66	(0.92,2.99)	0.092
Cluster III	−10.39	1.32	<0.001	2.57	(1.79,3.68)	<0.001
Cluster IV	8.77	2.56	0.001	0.46	(0.19,1.15)	0.096
	Sum of weights = 86,652,073			Sum of weights = 86,652,073		
	F Value = 49.17, R^2^ = 0.067			F Value = 27.59		

The linear regression represents the eGFR in each of the metabolic clusters. This model is an intercept (reference), and beta values (b) are reported in mL/min/1.73 m^2^; SE_b_ is the standard beta error. The logistic regression represents the odds of CKD in each metabolic cluster. This model presents odds ratios (OR) and 95% confidence limits (95% CL) where Cluster I is the referent group, and the reference value is 1.0. *p*-values were considered statistically significant at α = 0.05. The sum of weighted observations in this subsample was 86,652,073.

**Table 4:** Frequency of chronic kidney disease in metabolic clusters.

Cluster	CKD Cases Weighted Frequency	Total Weighted Frequency	CKD Cases in Cluster/Total CKD Cases (%, SE)	CKD Cases in Cluster/Total Cluster Sample (%, SE)	*p*-Value
I	2,317,090	21,412,722	13.91 (1.79)	10.82 (1.43)	<0.001
II	1,384,483	8,276,247	8.31 (1.89)	16.73 (3.70)	
III	12,782,719	53,790,449	76.76 (2.42)	23.76 (1.34)	
IV	168,245	3,172,655	1.01 (0.40)	5.30 (1.94)	

The frequency of chronic kidney disease is reported for each metabolic cluster. CKD was defined by an eGFR < 60 mL/min/1.73 m^2^ and/or an albumin to creatinine ratio (ACR) ≥ 30 mg/g [24]. The CKD Cases in Cluster/Total CKD Cases column represent the proportion of individuals with CKD divided by the total number of individuals with CKD across all clusters (n = 16,652,538). The CKD Cases in Cluster/Total Cluster Sample column represents the number of individuals with CKD in a cluster divided by the total number of individuals in the cluster. Abbreviations: CKD, chronic kidney disease; SE, standard error. The sum of weighted observations in this subsample was 86,652,073. The *p*-value indicates the probability of observing a difference in the number of CKD cases between the four clusters.

## Data Availability

All data are available from the CDC at https://wwwn.cdc.gov/nchs/nhanes/ (accessed on 30 August 2021).
